# Flexible and Transparent Polymer-Based Optical Humidity Sensor †

**DOI:** 10.3390/s21113674

**Published:** 2021-05-25

**Authors:** Katerina Lazarova, Silvia Bozhilova, Sijka Ivanova, Darinka Christova, Tsvetanka Babeva

**Affiliations:** 1 Institute of Optical Materials and Technologies “Acad. J. Malinowski”, Bulgarian Academy of Sciences, Akad. G. Bonchev str., bl. 109, 1113 Sofia, Bulgaria; 2 Institute of Polymers, Bulgarian Academy of Sciences, Akad. G. Bonchev Str., bl. 103-A, 1113 Sofia, Bulgaria; s.bozhilova@polymer.bas.bg (S.B.); sivanova@polymer.bas.bg (S.I.); dchristo@polymer.bas.bg (D.C.)

**Keywords:** flexible substrates, humidity, sensor, optical sensor, polymers

## Abstract

Thin spin-coated polymer films of amphiphilic copolymer obtained by partial acetalization of poly (vinyl alcohol) are used as humidity-sensitive media. They are deposited on polymer substrate (PET) in order to obtain a flexible humidity sensor. Pre-metallization of substrate is implemented for increasing the optical contrast of the sensor, thus improving the sensitivity. The morphology of the sensors is studied by surface profiling, while the transparency of the sensor is controlled by transmittance measurements. The sensing behavior is evaluated through monitoring of transmittance values at different levels of relative humidity gradually changing in the range 5–95% and the influence of up to 1000 bending deformations is estimated by determining the hysteresis and sensitivity of the flexible sensor after each set of deformations. The successful development of a flexible sensor for optical monitoring of humidity in a wide humidity range is demonstrated and discussed.

## 1. Introduction

Transparent and flexible sensing devices are of great interest because of their unique superior characteristics such as conformity, endurance and light weight. Sensors that can display induced color response, exhibit major flexibility and have a contactless method of detection are increasingly desired and studied mostly for monitoring ambient conditions and human health. Their reversibility and stability open the possibility for applications in human activity/health detection via optical humidity sensors. In general, depending on the sensing principle of the humidity sensor, there are different types of devices—capacitive, resistive, surface acoustic wave, those based on a change of color (colorimetric), etc. Among them, optical sensing, which detects alterations in environmental humidity via a color change, are particularly interesting due to the fact that it is not necessary for a specially trained specialist to use it, and its working principle presupposes an intuitive detection method [1,2]. Moreover, such a technology that exhibits an instantaneous reaction and does not need electricity could be applied in the form of flexible sensors for implantation in various types of clothing, masks, packaging, etc.

Diverse materials have been extensively studied in order to develop such low-cost humidity sensors with high performance and sensitivity, stability, a broad dynamic range and short response time. However, it is not easy to meet these requirements and the development of that kind of sensor faces multiple challenges such as finding compatible materials, suitable substrates and proper manufacturing techniques. A variety of materials can be used for deposition on flexible substrates [3,4,5,6] but the ones that stands out due to easy deposition in the form of thin films, relatively low cost, tailored functionality and fast response are polymers [7]. A number of different polymers, suitable for use as flexible substrates for sensor applications, are reported in the literature, including polyethylene naphthalate (PEN) [8,9], polyethylene terephthalate (PET) [10,11,12,13,14], polydimethylsiloxane PDMS [15,16], polyimide PI [17,18,19,20], polyvinyl alcohol (PVA) [21,22,23,24], polylactide (PLA) [25,26], polyurethane (PU) [27,28], polysulfone (PSU) [29], polyetheretherketone (PEEK) [30,31] and polycarbonate (PC) [32], along with other materials such as common paper [33,34], flexible glass [35] and last, but not least, composite [36] and multilayer substrates [37]. Among the most commonly used flexible substrates, PI provides a great solution for devices with higher annealing temperatures; however, its amber color makes it unsuitable for devices that require transparency. On the other hand, PET has high transmittance in the visible range around 88% [38,39] and despite its relatively low glass transition temperature of 70–80 °C, it is more suitable as a substrate in the design of flexible optical sensors. Besides the fact that PET substrates exhibit satisfactory optical properties in the visible range, they are known to be mechanically flexible under buckling or bending states. Finally, PET is interesting for implementation in sensors because it is compatible with spin-coating methods and has excellent moisture resistance and thermal stability, low cost and inertness of its surface. In light of these facts, the most common optical sensors are optical fiber-type temperature sensors and are included in humidity sensors, flexible UV exposure optical devices wrapped around fingers for monitoring skin health [40] and many more. Therefore, it will be an achievement to develop a flexible sensor that does not require a power supply, uses accessible technology like spin-coating and relies entirely on optical detection, for example, transmittance/reflection change or variation of the color.

In our previous studies [41,42], we have shown that poly (vinyl alcohol-*co*-vinyl acetal) (PVA-Ac) thin films with acetal content in the range of 18–28% are suitable for humidity sensing. The optimization of the chemical composition has revealed that thin films of PVA-Ac with 24% acetal content annealed at 60 °C exhibit the best performance. Further, various flexible substrates, including PET, PLA and composite polysiloxane *Compo-SiL^®^*, were employed to fabricate flexible devices for humidity detection [43]. The substrates were metalized with a gold/palladium layer prior to the deposition of the humidity-sensitive film for providing sufficient optical contrast of the devices, thus ensuring the highest sensitivity. In order to select the most suitable substrate, the optical and sensing properties of the obtained optical humidity sensors were thoroughly investigated and compared. The results demonstrated that the highest sensor sensitivity and reversibility were obtained when metalized PET was used as a flexible substrate [43].

In this paper, we concentrate our attention on polymer/PET devices and study the sensor’s performance after a series of bending deformations in the range of 25–1000. The surface morphology along with optical and sensing properties of the PET device are explored after each set of bending deformations. Furthermore, the bending effect on hysteresis, sensitivity and detection resolution is evaluated and the suitability of the flexible device for humidity monitoring by optical read-out is discussed.

## 2. Materials and Methods

Amphiphilic PVA-Ac copolymer with 24% acetal content was synthesized by hydrophobic modification of PVA (Fluka; 98% hydrolyzed; average polymerization degree 1600) with acetaldehyde (Fluka; ≥99%) as already described in [44,45]. Briefly, acetalization was carried out in aqueous solution of PVA of concentration 25 g·L^−1^. Acetaldehyde aqueous solution was added dropwise under vigorous stirring at a molar ratio of [acetaldehyde]:[PVA] = 1:4. The reaction was left to proceed under vigorous stirring at 30 °C for four hours. The product obtained was isolated from the reaction mixture by increasing the temperature to 60 °C, which led to copolymer precipitation due to its lower critical solution temperature (LCST) behavior. Copolymer was further purified by dissolution in deionized water at room temperature followed by precipitation at 60 °C, and finally dried in vacuum.

**Figure 1:**
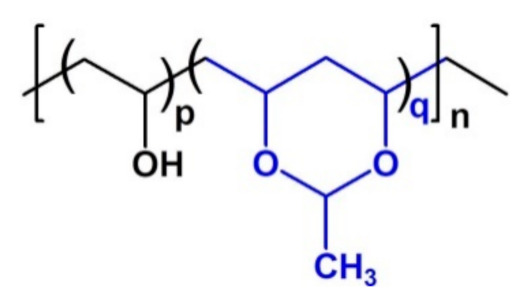
PVA-Ac copolymer structure.

The PVA-Ac copolymer structure is illustrated in Figure 1. Nuclear magnetic resonance (NMR) spectra were taken on a Bruker Avance DRX 250 (Billerica, MA, USA) spectrometer using dimethylsulfoxide-d_6_ as solvent. The content of acetal groups was estimated to be 24% from the ^1^H NMR spectrum of the copolymer (Figure S1) by comparing the integrated peak areas of the methine protons of PVA (peak 2 and 4) to the methine protons of acetal rings (peak 5).

The LCST behavior of the copolymer is known to be dependent on the extent of acetalization [45] and was evaluated by means of UV–Vis spectroscopy using a DU 800 Beckman Coulter (Brea, CA, USA) spectrophotometer. Transmittance at a wavelength of 500 nm of copolymer aqueous solution was studied as a function of temperature and the characteristic cloud point temperature of 30 °C was confirmed from the clouding curve (Figure S2).

Copolymer solutions of concentration 1 wt. % in mixed water–methanol solvent in a volume ratio of 20:80 were prepared and used for thin film deposition on PET substrates. Prior to the deposition, PET substrates were covered with a gold/palladium layer (Au/Pd ratio of 80:20 and thickness of 30 nm) through cathode sputtering for 60 s under vacuum 4 × 10^−2^ mbar using a Mini Sputter Coater SC7620 system (Quorum Technologies, Lewes, UK).

**Figure 2:**
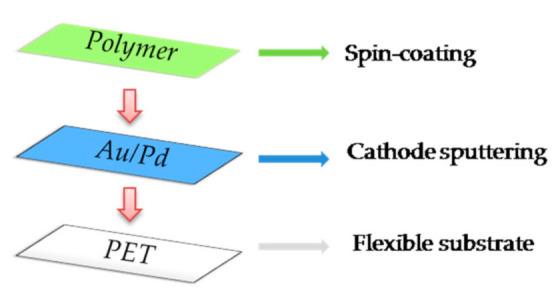
Schematic structure of the deposition of the individual elements of the PET/polymer sensor.

A spin-coating method was used to deposit thin polymer films on a flexible substrate. An amount of 0.250 mL of the solution was dropped on PET substrate and then spun stepwise. In the first step, the substrate was rotated for 1.5 s at 2500 rpm, and then in the second stage the rotations and duration were increased to 4000 rpm and 60 s, respectively. In both steps, the acceleration speed was 2500 rpm/s. Post-annealing of the samples was performed in air for 30 min at 60 °C. The schematic sequence of the film deposition method of the individual elements of the sensor is shown in Figure 2.

The surface morphology of the films was characterized by a Philips 515 scanning electron microscope.

Reflectance spectra of the films deposited on Si substrate were measured with a UV–Vis–NIR spectrophotometer (Cary 5E, Varian, Australia). In order to calculate refractive index (n), extinction coefficient (k) and thickness (d) of the films, nonlinear curve-fitting algorithms were used for minimization of the discrepancies between measured and calculated reflectance spectra [46].

The bending test was performed by a computerized homemade bending setup, with ESP301 control platform. During the bending test, the flexible substrate was fixed between two clamps horizontally and then was bent by pushing the two clamps together up to a bending radius of 4.37 mm. After each set of bends, the surface roughness and the optical quality of the deposited films were examined with a 3D optical profiler (Zeta-20, Zeta Instruments).

All humidity experiments were conducted by using a homemade bubbler system that generates vapors from liquids and samples were placed in a cell with a reference humidity sensor integrated into it [47]. Sensing properties of the films were studied by measuring transmittance as a function of humidity that gradually changed from 5 to 95% RH. Measurements took place at a fixed wavelength that was preliminarily chosen as the wavelength of the highest humidity responses, i.e., transmittance change ∆*T_max_* = |T_95RH_–T_5RH_|. The percentage of hysteresis (*H*) was calculated from the following equation:

(1) $$H\left(\%\right)=\frac{max\left|T_{up-}T_{down}\right|}{\Delta T_{max}}.\frac{\Delta RH_{hyst}}{RH2-RH1}.100$$

where *T_up_* and *T_down_* are transmittance values measured when humidity increases and decreases, respectively; ∆*T_max_* is the maximum transmittance change in the studied humidity range (RH2-RH1) and ∆*RH_hyst_* is the humidity range where hysteresis is observed. The sensitivity of the sensor *S* was calculated according to the following equation:

(2) $$S=\frac{\Delta T}{RH2-RH1}$$

where ∆*T* is the change in the film’s transmittance in % for humidity variation from RH1 to RH2.

The accuracy/resolution (Δ*RH*) of detection depends on the sensitivity and measurement accuracy in the signal and was calculated as:

(3) $$\Delta RH=\frac{errT\;\left(\%\right)}{S\;\left(\%\right)}$$

where *err*T = 0.1% is the experimental error (accuracy) of *T* and *S* is the sensitivity, calculated by Equation (2). Accuracy and sensitivity are calculated for two different humidity ranges: 5–75% and 75–95%.

## 3. Results and Discussion

### 3.1. Optical Properties and Surface Morphology

**Figure 3:**
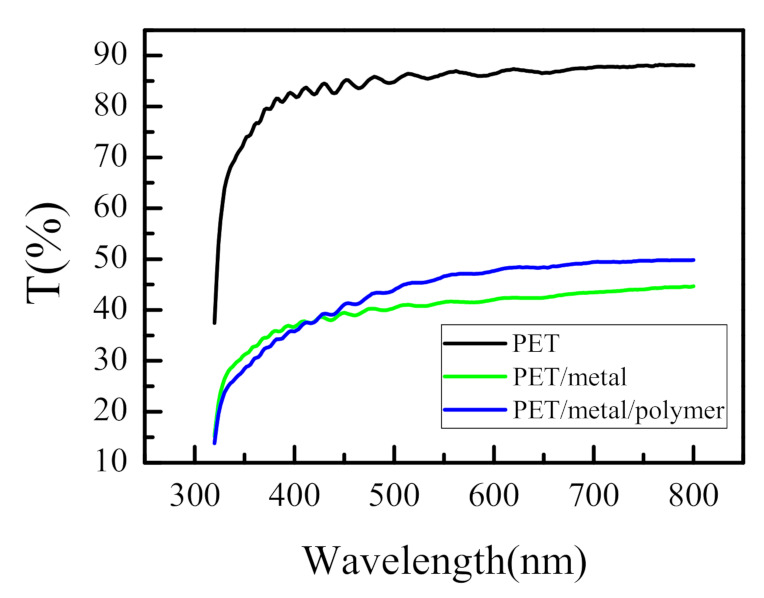
Measured transmittance spectra of bare PET substrate, PET substrate covered with Au:Pd layer and metalized PET covered with polymer film.

Transmittance spectra of bare and metalized PET substrate, as well as spectra of the metalized PET covered with polymer film, are shown in Figure 3. It is seen that PET substrate is transparent in the visible spectra range and has transmittance values of more than 80% for wavelengths higher than 380 nm. Interference peaks with a small amplitude are observed in the spectra, mostly pronounced for the bare PET substrate. Considering that the thickness of the substrate is 75 µm, it is clear that the origin of these peaks cannot be the multiple reflections from the front and back sides of the substrate, firstly because their amplitude is very low and secondly because the distance between two adjacent peaks is too large. Our additional calculations have shown that an area with an approximate thickness of 2 µm exists on top of the substrate with a refractive index slightly higher than the refractive index of the PET substrate. Due to the difference in refractive indices of the substrate and the surface area, multiple reflected waves from the top and bottom boundaries of the area occur. Owing to the small thickness (approximately 2 µm), these waves are coherent and interfere constructively and destructively depending on the wavelength, resulting in the observed transmittance maxima and minima.

It is also seen from Figure 3 that when covered with a metal layer, substrate transmittance decreases almost twice in the visible spectral range due to the absorption of the Au:Pd layer. Despite this drop, the level of transmittance stays sufficiently high (more than 40%) and good sensitivity of detection is expected.

We should mention here that if a non-metalized substrate is implemented for detection instead of a metalized one, the optical contrast, i.e., the difference between the refractive indices of the sensitive medium and substrate, will be very low. This will lead to low sensitivity of detection because it will be difficult to distinguish the thin film from the substrate.

It is seen from Figure 3 that the deposition of polymer film does not substantially change the spectra of metalized PET.

**Figure 4:**
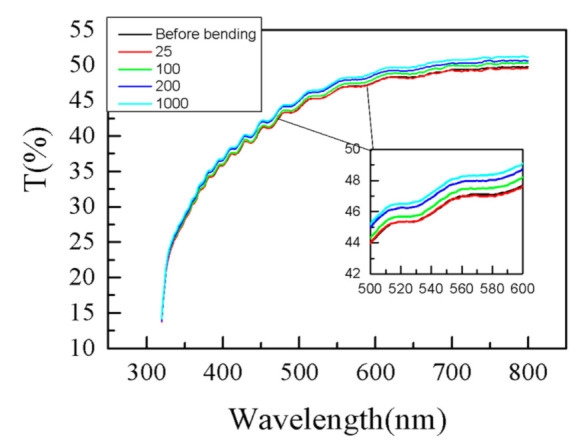
Measured transmittance spectra T of metalized PET substrate covered with polymer film before bends and after each set of deformations.

The impact of bending deformations on transmittance spectra T of the sensor is illustrated in Figure 4 where T spectra, after each set of bending deformations, are displayed along with T spectrum before bending. It is seen that the transmittance value gradually increases after each set of bends and its value at 600 nm changes from 47.6% before bending to 49.1% after 1000 deformations.

The possible reason for the observed slight increase in T is the change in transmittance of the metal overlayer due to fine sub-micron cracks generated as a result of bending deformations. Further, it is seen from Figure 4 that the wavelength position of interference peaks and their number are the same for all bending deformations. Considering that the wavelength position of the peak is extremely sensitive to the thickness change, we may conclude that bending deformations and inevitable stretching do not lead to substrate thickness changes, for example, thinning due to stretching.

**Figure 5:**
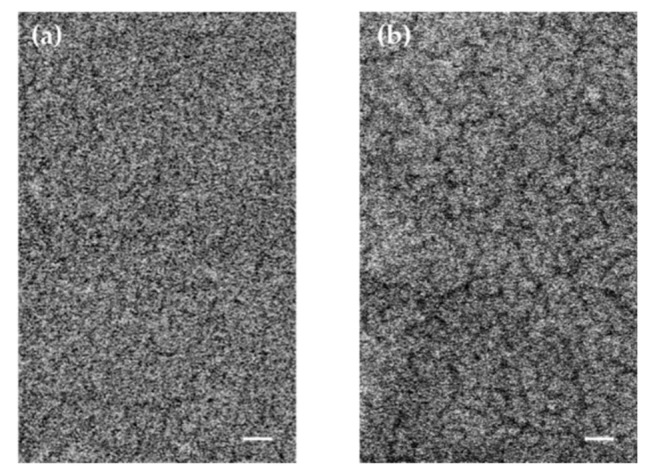
SEM micrographs of the surface of the metallized PET substrate before (**a**) and after 1000 (**b**) bending deformations. The bar is 100 nm.

The assumption of the generation of fine cracks in the metal film is confirmed by the comparison of surface morphology of metalized PET substrate before bending and after 1000 bends, as presented in Figure 5. In the case of bent substrate (Figure 5b), a network of fine cracks is easily seen that is responsible for the slight increase in transmittance.

**Figure 6:**
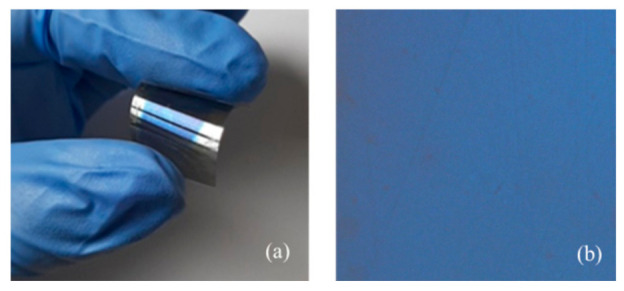
Picture of the bent sensor (**a**) and optical image of the surface of the PET/polymer sensor after 1000 bending deformations at magnification of 100× (**b**).

In order to explore the compatibility and suitability of the selected materials (PET and hydrophobically modified PVA), optical images of the surface of the PET/polymer sensor were taken using an optical profiler. The effect of bending on surface morphology was established after each set of bending deformations (Figure 6). Figure 6 shows the picture of the sensor when bending deformation is applied and its surface at a magnification of 100× after 1000 bends.

It is clearly seen that after 1000 bending deformations, the surface of the sample is still smooth and free from cracks and defects. Based on the pictures in Figure 6, we came to the conclusion that the film is very homogenous across its thickness and across the entire surface of the substrate due to the uniform color of the optical image (Figure 6b). The optical profiler presents the surface in real color and each deviation of the background means the existence of areas with different thicknesses and/or refractive indices. Thus, the uniform color of the picture is associated with uniform thickness and refractive index of the film. The inspection of the initial surface (not shown here) demonstrates high similarity to the sample surface after bending. This result is not surprising and explains almost the same transmittance values obtained after bending deformations.

### 3.2. Humidity Sensing Experiments

**Figure 7:**
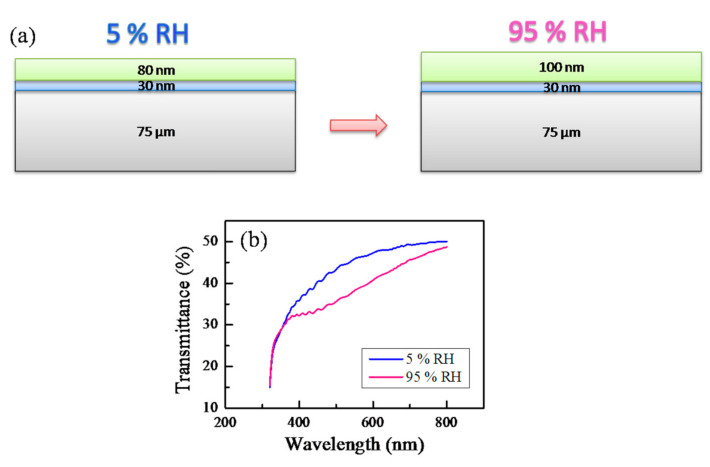
Scheme of the effect of the humidity on the sensor’s structure (**a**) and transmittance spectra at low and high humidity levels (**b**).

All humidity sensing experiments were conducted by using a homemade bubbler system that generates vapors from liquids. The tested samples were placed in a cell with a reference humidity sensor integrated into it. The first transmittance measurement was performed in air. Then, dry argon was purged in the cell. As a result, the relative humidity in the cell gradually changed from the RH of the air (usually 45–55% RH) to 5% RH (i.e., the first end point) and the transmittance spectrum at low humidity was measured (Figure 7). In the next step, the cell was connected to a homemade bubbler system using valves and water vapors from water were generated and delivered to the cell using argon as a carrier gas. As a result, the humidity in the cell increased from 5% RH to 95% RH (the second end point) and transmittance spectra at a relative humidity of 95% RH were recorded (Figure 7). The relative humidity in the cell was measured continuously with steps of 1 s by the reference humidity sensor that was located in the cell close to the sample under study. It is known from our previous experiments [41,42,43,45] that due to exposure to humidity, the polymer swells and an increase in the thickness and a decrease in the refractive index of the polymer film occur. As a result, the transmittance spectrum shifts toward longer wavelengths (Figure 7b) and a change in the color of the sensor can be expected.

**Figure 8:**
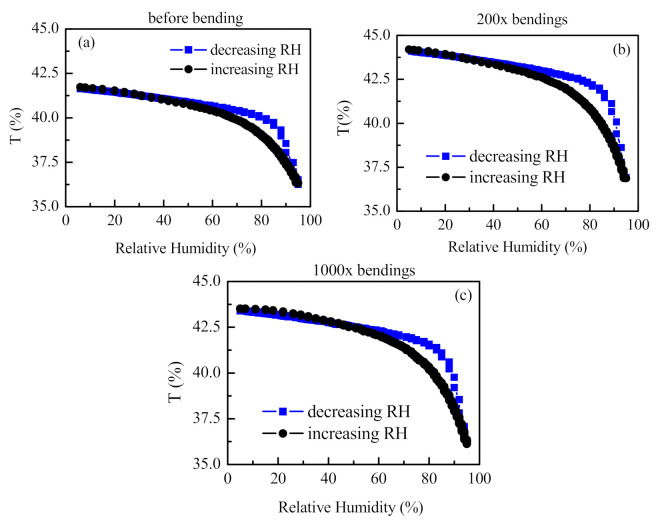
Dependence of transmittance T on relative humidity before (**a**), after 200 (**b**) and after 1000 (**c**) bending deformations.

In order to continue the detailed study of the impact of bending on the humidity-sensing ability of the samples, transmittance values (T) were monitored as a function of relative humidity (RH) that gradually changed in the range from 5 to 95% RH. The plots of T versus RH were further used to evaluate the hysteresis values H, transmittance changes ∆*T*, accuracy/resolution Δ*RH* and sensitivity S of the sensor before and after all sets of bending deformations. Figure 8 presents the T vs. RH loops before bending deformation and after sets of 200 and 1000 bends.

**Figure 9:**
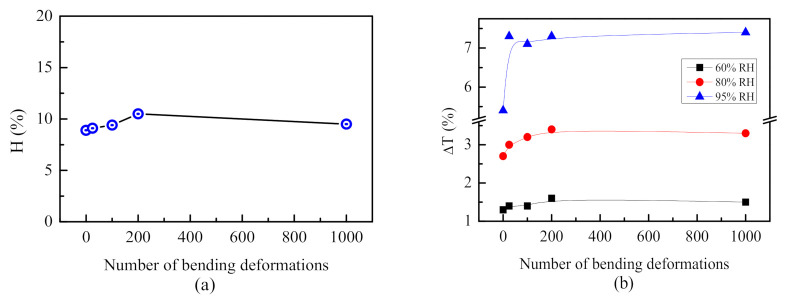
Percentage of hysteresis, H calculated with Equation (1) (**a**) and transmittance change ∆*T_max_* (%) at different levels of humidity (**b**) for PET/Au:Pd/polymer sensor calculated before and after 25, 100, 200 and 1000 bending deformations.

It is seen from Figure 8 that the linear dependence of T vs. RH in humidity ranges from 5 to 75% RH and 75–95% RH observed for the sensor before bending is preserved after 1000 bends. Surprisingly, the sensitivity, which expresses itself through the slope of the plot, increases after bending deformations. The enhancement of sensitivity is more pronounced for higher humidity levels. In the humidity range of 5–75%, S increases from 0.03 to 0.04 and leads to an improvement in the resolution from 3.3% RH to 2.5% RH. For higher humidity levels (>75% RH), the effect of bending is more significant: S increases from 0.17 to 0.24, so the sensor can resolve 0.6% RH and 0.4% RH, respectively, when measuring high humidity levels (RH > 75%) (Table 1). Further, using Equation (1) and the data from hysteresis loops presented in Figure 8, we calculated the percentage of hysteresis before and after bending deformations and the data are presented in Figure 9a along with the data for transmittance change ∆*T_max_* = T_95%_ − T_5%_ (Figure 9b).

Figure 9 demonstrates the same trend of hysteresis and sensitivity with bending deformations: both parameters increase at the beginning of the bending test and then reach steady state. It is seen from Figure 9b that the effect of bending deformation on ∆*T_max_* is stronger for higher humidity levels: ∆*T* after 1000 bends increases by 15%, 22% and 37% for humidity levels of 60%, 80% and 95%, respectively, as compared to ∆*T* before bending; therefore, the conclusion could be made that the enhancement of the sensitivity is most pronounced at higher humidity levels. We could assume that the bending deformations decrease the adhesion forces between the metalized PET substrate and the polymer thin film to some extent, thus improving the polymer swelling. The effect is more pronounced for higher humidity levels because the swelling is stronger.

In Table 1, the percentage of hysteresis H, transmittance changes ∆*T*, accuracy/resolution Δ*RH* and sensitivity S of the sensor before and after 25, 100, 200 and 1000 bending deformations are summarized.

According to the comparison of our flexible polymer sensor with similar sensors, we should mention that the most sensitive technology for optical humidity sensing is fiber optic sensing. The recent achievements in polymer optical fiber sensors have shown that the sensitivity can reach 0.07 nm/% RH if neat PVA is implemented as sensitive media, while an enhancement of sensitivity to 0.1 nm/% RH is obtained for PVA doped with graphene quantum dots [23]. In order to make a comparison with our sensor, we calculated the resolution using Equation (3) and obtained values of 0.3% RH and 0.2% RH, respectively. It is seen from Table 1 that the accuracy of 0.4% RH and 0.6% RH achieved by our sensor is comparable to the highest accuracy reached to date with PVA-based sensitive materials. However, in our case, the two serious drawbacks of optical fiber technology have been overcome. These are the relatively high cost of optical equipment, particularly spectrometers and optical spectrum analyzers, and the complicated preparation of sensitive elements, especially in the case of side-polished optical fibers. It is interesting to note that similar resolutions of 0.25% in the 5–55% RH range and 0.10% in the 55–90% RH range were obtained for respiration monitoring using surface acoustic wave technology and three-dimensional architecture graphene/PVA/SiO_2_ layered sensing film [48].

## 4. Conclusions

The development of a flexible and transparent polymer-based optical humidity sensor is demonstrated. The sensor consists of pre-metallized PET substrate covered with a spin-coated thin film of poly(vinyl alcohol-*co*-vinyl acetal) with acetal content of 24% and a thickness of 80 nm. The metallization of PET substrate provides sufficient optical contrast between the humidity-sensitive film and PET substrate while the small thickness assures a fast response. A small increase of 1.5% of transmittance of the sensor is obtained after 1000 bending deformations, mostly due to the fine network of cracks appearing in the metal film as a result of bending. However, it is demonstrated that the influence of up to 1000 bending deformations on the surface morphology and optical quality of the sensor is negligible. Moreover, an enhancement of sensitivity of more than 40% is observed after bending for relative humidity higher than 75%, resulting in an improvement in resolution from 0.6% RH to 0.4% RH. It is assumed that the weakening of adhesion due to bending is the most probable reason, thus facilitating polymer swelling. The results obtained open up new opportunities for the use of PET/Au:Pd/polymer structures as in situ optical flexible sensors for humidity detection.

## Figures and Tables

**Table 1:** Percentage of hysteresis H in %, transmittance changes ∆T in %, accuracy/resolution ΔRH in % RH and sensitivity *S* in %/% RH.

Variable/ Number of Bending Deformations		Before Bending	25	100	200	1000
H (%)		8.9	9.1	9.4	10.5	9.5
Δ*T* for 95% RH		5.4	7.3	7.1	7.3	7.4
Δ*RH*	Range 5–75% RH	3.3	5	2.5	2.5	2.5
	Range 75–95% RH	0.6	0.6	0.4	0.5	0.4
S	Range 5–75% RH	0.03	0.02	0.04	0.04	0.04
	Range 75–95% RH	0.17	0.18	0.23	0.22	0.24

## Data Availability

Not applicable.

## Supplementary Materials

The following are available online at https://www.mdpi.com/article/10.3390/s21113674/s1, Figure S1: ^1^H NMR (250 MHz; solvent DMSO-d_6_) spectrum of PVA-Ac (A) compared to the spectrum of starting PVA (B).; Figure S2: Clouding curve of PVA-Ac aqueous solution of concentration 5 g·L^−1^. The cloud point is counted at the inflection of transmittance-vs-temperature curve.
