# Supplementary material for: Flexible and Transparent Polymer-Based Optical Humidity Sensor
Source: Sensors (Basel). 2021 May 25;21(11):3674. doi: 10.3390/s21113674 (PMC8198816; doi:10.3390/s21113674)
Supplement: Supplementary file 1 [file sensors-21-03674-s001.zip › sensors-1202008-SI.pdf]

Supplementary materials

Flexible and Transparent Polymer-Based Optical Humidity Sensor †

Katerina Lazarova [1,*], Silvia Bozhilova [2], Sijka Ivanova [2], Darinka Christova [2] and Tsvetanka Babeva [1,*]

[1] Institute of Optical Materials and Technologies ''Acad. J. Malinowski'', Bulgarian Academy of Sciences, Akad. G. Bonchev str., bl. 109, 1113 Sofia, Bulgaria; klazarova@iomt.bas.bg (K.L.); babeva@iomt.bas.bg (T.B.);

[2] Institute of Polymers, Bulgarian Academy of Sciences,Akad. G. Bonchev Str., bl. 103-A, 1113 Sofia, Bulgaria; s.bozhilova@polymer.bas.bg (S.B.); sivanova@polymer.bas.bg (S.I.); dchristo@polymer.bas.bg (D. Ch.);

* Correspondence: klazarova@iomt.bas.bg (K.L); babeva@iomt.bas.bg (T.B); Tel.: +359 02-979-3521 (K.L.)

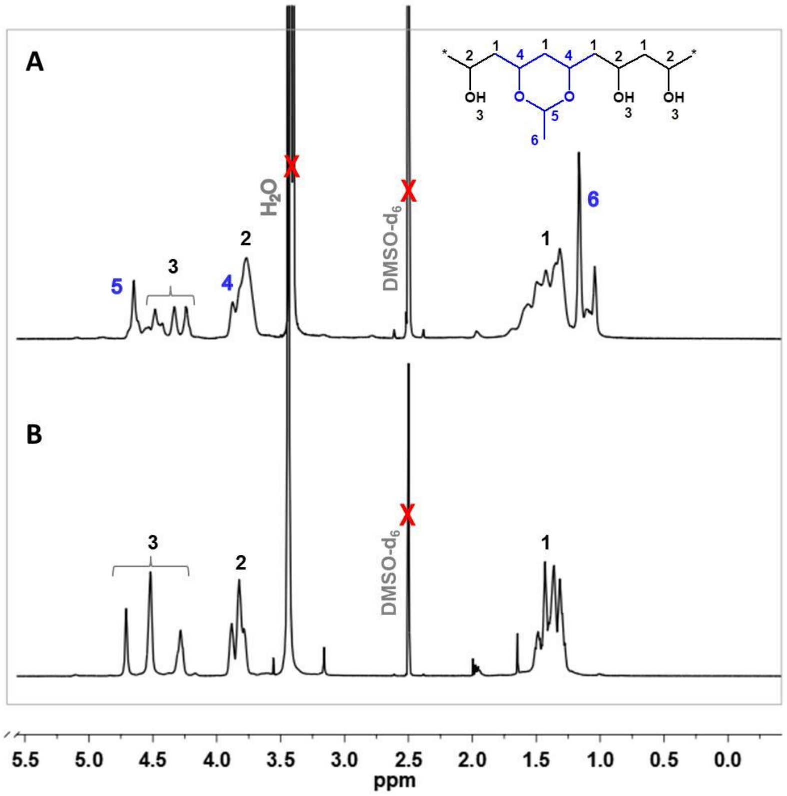

Figure S1. 1H NMR (250 MHz; solvent DMSO-d6) spectrum of PVA-Ac (A) compared to the spectrum of starting PVA (B).

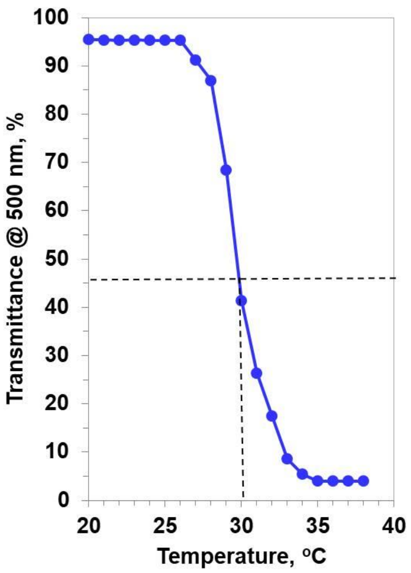

Figure S2. Clouding curve of PVA-Ac aqueous solution of concentration 5 g.L-1. The cloud point is counted at the inflection of transmittance-vs-temperature curve.
